# Supplementary material for: Community risk perception and barriers for the practice of COVID-19 prevention measures in Northwest Ethiopia: A qualitative study
Source: PLoS One. 2021 Sep 24;16(9):e0257897. doi: 10.1371/journal.pone.0257897 (PMC8462701; doi:10.1371/journal.pone.0257897)
Supplement: S1 File — (PDF) [file pone.0257897.s001.pdf]

I. Qualitative study Information Sheet for participants of in-depth interview and Focus group discussions in South Gondar administrative zone, Northwest Ethiopia

Hello! dear participant!

We are carrying out a study on "**Community Risk perception and Barriers for the Practice of COVID-19 Prevention Measures in Northwest Ethiopia: A Qualitative Study"** Therefore inorder to achieve this objective, having an in-depth interview and focus group discussion with you has become essential. We hope that the interview and the discussions we would be having with you are very much helpful for the community and government bodies to explore the barriers for practicing RECOMMENDED prevention methods of COVID-19 which will be used to make intervetions by developing different intervention startegies and polices. We would like to tell you that you are selected purposely to be the participant of this study.

Objectives of the study: To explore the Community Risk perception and perceived Barriers for the practice of COVID-19 Prevention Measures in South Gondar zone, Northwest Ethiopia.

Benefits of the study: The study will explore the Community Risk perception and the barriers of the community members for not practicing RECOMMENDED prevention methods of COVID-19 global pandemic and the findings will be used to take appropriate intervention on tackling the problem in the area in particular and in the country as a general.

Risk of the study: Participating in this study did not have any risk or harm to participants of the study.

Costs to the participants: Participation in this research is free and it does not cost the participants anything.

Rights of the participants: participation in this research is entirely of participants own free will, and they are free to withdraw at any time during the study without offering any reasons why. They may respond to the questions or they may not answer to questions they do not want to and they may end the interview at any time they want. Participants can ask any question that is not clear to them.

Confidentiality: All information provided in this study will be confidential. All forms will be coded and information will be entered into password protected computers. We will do all that is in power to ensure that the participants identity and the information that they have provided is kept confidential.

II. Informed consent form for in-depth interview participants

As with the information given above, participation in this in-depth interview is voluntary and has no any risk. Your answers will remain confidential, and we will not be taking down your name or address, so your answers will be anonymous. You can choose not to answer any individual question that you do not want to answer and you may end this interview at any time you want. However, we hope that you will participate in this in-depth interview since your honest response to the interviews will be essential for the study.

At the same time, we would like to appreciate your voluntarily participation in the study after a thorough understanding of the information given to you.

Now, are you willing to participate in this study?

1. No (say thank you)
2. Yes (continue interviewing)

Name of principal investigator: Aragaw Tesfaw

Cell phone: 0921743820

Name of Administrative Woreda_________________________

Name of interviewer______________________ signature__________

Date of interview _____/_____/_______in Ethiopian calendar

In doing this interview, we will raise some questions concerning the Community Risk perception and perceived Barriers for the practice of COVID-19 Prevention Measures.

III. Informed consent form for Focus Group Discussion participants

As with the information given above, participation in this focus group discussion is voluntary and has no any risk. Your answers will remain confidential, and we will not be taking down your name or address, so your answers will be anonymous. You can choose not to answer any individual question that you do not want to answer and you may end this discussion at any time you want. However, we hope that you will participate in this Focus group discussion since your honest participation with other community members will be essential for the study.

At the same time, we would like to appreciate your voluntarily participation in the study after a thorough understanding of the information given to you.

Now, are you willing to participate in this focus group discussion?

3. No (say thank you)
4. Yes (Join the participants to the group for Focus Group Discussion)

Name of principal investigator: Aragaw Tesfaw

Cell phone: 0921743820

Name of Administrative Woreda__________________________

Name of FGD Facilitator _______________________ signature__________

Date of Focus group discussion _____/_____/_______in Ethiopian calendar

In doing this focus group discussion, we will raise some questions concerning the Community Risk perception and perceived Barriers for the practice of COVID-19 Prevention Measures in this locality
