# Supplementary material for: Community risk perception and barriers for the practice of COVID-19 prevention measures in Northwest Ethiopia: A qualitative study
Source: PLoS One. 2021 Sep 24;16(9):e0257897. doi: 10.1371/journal.pone.0257897 (PMC8462701; doi:10.1371/journal.pone.0257897)
Supplement: S2 File — (PDF) [file pone.0257897.s002.pdf]

I. **In-depth interview Guide (discussion points with community key informants)**

1. Would you please tell me your age, educational status, occupation, marital status, and how long you have been living here? No need to mention your name.
2. In your opinion, what do you think about the community understanding about Corona virus ?

   Probing questions

   - Severity of the disease
   - Mode of transmission
   - clinical features and
   - Knowledge and practice of prevention methods
   - Do you think as you could be infected by this disease? How about the community?
3. What did you feel if you notice the features of COVID -19 ?

   Probing questions

   - To whom you first tell your problem,
   - What solutions you made to manage the changes in your body, mainly respiratory system,
   - Would you like to use traditional treatments?
4. What do you think about the barriers /challenges to practice COVID-19 prevention measures in the community?

   Probing questions

   - Personal related barriers
   - socio-cultural barriers
   - Religious beliefs
   - economic barriers ,
   - Community trust about COVID-19 medical care
5. What do you think about the impact of COVID-19 ?

   Probing questions

   - social impact
   - political and economical effects

6. Do you think our health care system is an adequate enough to control COVID-19 pandemic?
7. Do you have anything to share with me which is not raised in the discussion before I end the interview?

Finally, I would like to express my heartfelt thanks for your voluntary participation in this in-depth interview.
