# Supplementary material for: Community risk perception and barriers for the practice of COVID-19 prevention measures in Northwest Ethiopia: A qualitative study
Source: PLoS One. 2021 Sep 24;16(9):e0257897. doi: 10.1371/journal.pone.0257897 (PMC8462701; doi:10.1371/journal.pone.0257897)
Supplement: S3 File — (PDF) [file pone.0257897.s003.pdf]

I. **<u>Focus Group Discussion Guide (discussion points with community members)</u>**

1. Would you please tell me your age, educational status, occupation, marital status, and how long you have been living here? No need to mention your name.
2. In your opinion, how do you understand about Corona virus ?

   Probing questions
   - Severity of the disease
   - Mode of transmission
   - clinical features and
   - Knowledge and practice of prevention methods
3. What did you feel if you notice the features of COVID -19 ?

   Probing questions
   - To whom you first tell your problem,
   - What solutions you made to manage the changes in your body, mainly respiratory system,
   - Would you like to use traditional treatments?
4. Do you think Ethiopians will be affected by COVID-19 like the most affected countries in the world? Why?

   Probing questions
   - Do you think as you are at risk of COVID-19?
   - Do you fear of acquiring the infection?
   - How the community thinks about COVID-19?
5. Do you thing COVID-19 is God penality do with our sins? Why, how?
6. What do you think about the barriers /challenges to practice COVID-19 prevention measures in your community?

   Probing questions
   - Personal related barriers
   - socio-cultural barriers
   - Religious beliefs
   - economic barriers ,
   - Community trust about COVID-19 medical care
7. What do you think about the impact of COVID-19 ?

Probing questions

- social impact
- political and economical effects
- Impacts on family and nation

8. Do you have anything to share with me which is not raised in the discussion before I end the interview?

We would like to express our heartfelt thanks for your voluntary participation in this focus group discussion.
