# Supplementary material for: Community risk perception and barriers for the practice of COVID-19 prevention measures in Northwest Ethiopia: A qualitative study
Source: PLoS One. 2021 Sep 24;16(9):e0257897. doi: 10.1371/journal.pone.0257897 (PMC8462701; doi:10.1371/journal.pone.0257897)
Supplement: S4 File — (PDF) [file pone.0257897.s004.pdf]

Table 2: Themes and categories (subthemes) developed from the narrations of In-depth Interview (IDI) and Focus Group Discussion (FGD) participants, South Gondar Administrative zone, North-west Ethiopia, 2020

Themes	Categories/sub-themes)	Narrations from both FGD & IDI participants (selected quotes/main findings)
Personal related barriers	Lack of information access	- *"I know other disease like TB, AIDS but I did not hear about this new disease which you called Corolla or Corona. I heard it know from you. As you know, my house is far from the health post. Unless the health extension worker comes to our Kebele (lower administrative area), I could not hear anything about health. The health extension workers were educating me about TB and HIV last year. I could not get her in the last two months. " (A 60 years old female IDI participant)*<br>- *"Okay, I heard the name of the disease which is called "Corona'. I heard from television news. I did not know about its characteristics. There was no such disease in our country. It is outside our country. I think it is in China " (A 52 years old female FGD participant)*<br>- *"ohhh… I do not know Corona. I heard this new disease from the health extension worker, However, I did not understand about the disease. I do not know, it will infect me or not I heard as most people said, it is highly transmitted from person to person. I did not see any patients with this disease, but there is one person who sent to quarantine suspected for this disease". (A 32 years old female FGD participant)*
	Lack of knowledge and awareness about COVID-19 infection	- *"I know this disease when people talk and it is now a public agenda. I heard the name Corona virus disease through the telecom messages in my phone. However, I did not understand about the prevention methods. People say eating onions and honey is good but I did not practice it" (A 45 years old IDI participant)*

		- *"I heard the name corona virus in Ethiopian television. I have television and I always heard news and I heard the prevention methods of corona virus but I am not aware and clear about its importance." (A 35 years old IDI participant)*<br>- *"I did not know How Corona is transmitted from person to person. I did not understand the disease. I do not know the symptoms of the disease." A 28 years old FGD participant*
Socio-demographic and economic barriers	Socio-demographic barriers	- *"My child is always told me about this disease. He is freighted for me. As he told me, the disease is fatal for old people like me. I am 70 years old, and I have heart problems. My child is a college student and he told me as I am at risk of this disease if it comes to our village …".A 70 years old FGD participant*<br>- *"I am a farmer. I live in a very remote areas. Unless I come to a health facility, no one can come to my village and tell me about the disease since there is not transport access to our village. I and my family did not think too much about Corona." (A 46 years old FGD participant).*<br>- *"--- I think the disease does not affect young people. Therefore I will not be at risk of this disease. So I do not afraid and care about it too much" A 24 years old FGD participant*<br>- *"I lived in a rural area so I faced many difficulties when I came to this now because of the long distance. Since there is no road transport access. Thus I could not get information's easily about the disease. I heard only from kebele leaders when they told us to wash our hands and they warned us to avoid social gatherings" (A 52 years old participant)*

	Socio-economic barriers	- *"I heard that one of the prevention methods is stay at home. However, I could not stay at home because I could not get my daily foods because I am a daily worker." A 28 years old FGD participant*<br>- *"I think stay at homes very difficult for most people in our country since most people could not get their daily consumption, rather the impact will be worse than the effect of the disease. hahaha no, no, I could not stay at home" (A 46 years old IDI participant)*<br>- *"I have financial problem. I could not get adequate soap for my family to regularly wash our hands. It is costly for me and I could not afford to buy it. I do not know hand sanitizer, I only heard from, you know. Is it available at the market? If the government provides to us, it will be good" (A 39 years old FGD participant)*<br>- *"I think soap and sanitizer is not accessible for all people. Most of us have low economic status and we have difficulties to get it unless the government give us. so we could not use it frequently." (A 40-year-old FGD participant said)*
Cultural and religious related reasons	Cultural related reasons	- *"People say different things about corona virus. As I heard from other people, corona will infect those who eat wild animals but not us. In our culture, it is not allowed to eat wild animals, so I think the disease could not infect us. GOD will keep us. " (A 38 year old IDI participant)*<br>- *"…the people in my community believe that the disease does not come from our village and they strongly recommend the government to control those who come from abroad." (A 57 years old participant)*<br>- *"In our community we have a strong social interaction and most of the time we live together and we meet for different social gatherings like mourn, wedding, Idir, Ikub and even we drink coffee*

		together. In addition, we do not want to eat alone, we eat together by sharing what we have in our hands, so this is very challenging to stop in a short periods of time." (A 64 years old participant)<br>- *"I think it is very challenging to practice the prevention measures* because our way of living is difficult to bring a behaviour change. If someone tries to practice, some others are so careless. Thus one practice may not be enough and does not give a guarantee unless all other people surrounding you practice it. Some people do not fear the disease and they are so careless to *practice prevention measures."* (A 63 years old participant)<br>- *"As I think, most of us try to practice, but sometimes we forget it and we do our normal life since we do not have such culture before."* (A 50 years old participant)<br>- *"As you know we live in hot area and the people in my village believe that the disease cannot come to hot areas, the disease cannot survive in hot areas…" (A 26 years old participant)*<br>- *"As I heard the disease transmission will be fast* in cold environment than hot areas. We are living *in in a hot climatic areas, so the disease will not affect us that much." (A 40 years old IDI* participant)<br>- *"I think the herbal medication which we used for common cold will be effective to treat corona since it has similar characteristics."* (A 40-year-old *FGD* participant said)<br>- *"If the virus infects me, I will go to use holy water and I will pray. I will not prefer to go to the health facilities since there is no any medical treatment till know."* (A 45-year-old participant said).

	Religious related reasons	- *"Please trust GOD, if you strongly believe in the power of GOD, this disease will not infect you. I do not afraid about this disease but I will try to practice the prevention methods as much as I can " (A 65 year's old FGD participant)*<br>- *"I think this disease is due to our sin so praying and going to use holy water is the only option we have to do". (A 68 year's-old IDI patient) "Peoples may have different sayings about this disease. I am sure that this disease is due to our sin and GOD wishes to teach us by this disease and he just shows his supreme power to the world as no one can do anything without the will of GOD. As my friend says, GOD will protect us from this disease". (A 50-year-old participant IDI said)*<br>- *"There were several predictions that this kind of epidemic will occur from our ancestors. Thus, the disease is due to in human acts like homosexuality, racism. Currently there are a lot of immoral acts conducted in our country which leads us to be punished by GOD" (A 55 year's old IDI participant)*<br>- *"As you know we all have religion and most of our peoples are also followers of either Orthodox or Muslim religion. We trust GOD, so we should not have too much worry about this disease, GOD will save us from this event. What expected from us is properly practice the rules of our religion and we have to pray day and night" A 70 years old FGD participant*<br>- *"I am praying day and night for the health of me and my family and I strongly trust GOD, I do not think this disease will infect me." (A 38 year old* IDI *participant)*
