# Supplementary material for: Community risk perception and barriers for the practice of COVID-19 prevention measures in Northwest Ethiopia: A qualitative study
Source: PLoS One. 2021 Sep 24;16(9):e0257897. doi: 10.1371/journal.pone.0257897 (PMC8462701; doi:10.1371/journal.pone.0257897)
Supplement: Attachment — Submitted filename: Response to Reviewers.docx [file pone.0257897.s005.docx]

**Dear, Editor,**

**Greetings!**

Thank you very much for all the comments provided regarding our manuscript entitled “**Perceived Barriers for the Practice of Covid-19 Prevention Measures in Northwest Ethiopia: A Qualitative Study”** which are fully accepted and included in the revised version. I have accordingly made necessary revisions on the paper following the comments provided from the reviewers and editor. I also attached the themes and categories (Sub-themes) as supporting information file 4 and in-depth interview and Focus group discussion guides as supporting information file 2&3 for the reviewers based on their request. in addition I attached the study information sheet and consent form as supporting information file-1. For your kind consideration, please find a point by point response to the comments in the next page of this letter and a submitted new revised version of the manuscript.

All new changes have been highlighted in **dark blue** in the main document in order to facilitate review.

I hope that you will find the edits as per your expectation and I look forwards to hear from you
soon.

Yours Sincerely,

Aragaw Tesfaw (MPH, Epidemiology)

Lecturer, Department of Public health

College of health sciences, Debre Tabor University

Email: [aragetesfa05@gmail.com](mailto:aragetesfa05@gmail.com)

Phone: 251921743820

I. **Point by point responses to editor comments**
1. Please ensure that your manuscript meets PLOS ONE's style requirements, including those for file naming. The PLOS ONE style templates can be found at

<https://journals.plos.org/plosone/s/file?id=wjVg/PLOSOne_formatting_sample_main_body.pdf> and

<https://journals.plos.org/plosone/s/file?id=ba62/PLOSOne_formatting_sample_title_authors_affiliations.pdf>

- **Author Reponses**: corrected /edited based on the given editor comment and we prepared the manuscript based on the PLOS ONE author guide line.

2. We suggest you thoroughly copyedit your manuscript for language usage, spelling, and grammar. If you do not know anyone who can help you do this, you may wish to consider employing a professional scientific editing service.

- **Author Reponses**: We invited English language editor for grammar edition and she corrected grammatical issues accordingly based on the recommendation. in addition we used on line grammar editing services.

Upon resubmission, please provide the following:

- The name of the colleague or the details of the professional service that edited your manuscript

**Author Reponses**: the name of the colleague who edit the grammar was Emaway Belay, she is health education, promotion and Behavioral science expert and she has master of public health

- A copy of your manuscript showing your changes by either highlighting them or using track changes (uploaded as a *supporting information* file)

**Author Reponses**: All new changes have been highlighted in **dark blue** in the main document and other requested information’s are uploaded as supporting information files

- A clean copy of the edited manuscript (uploaded as the new *manuscript* file)

**Author Reponses**: The edited manuscript is uploaded as Manuscript file

3. Please specify in your ethics statement:

a) Whether the ethics committee approved the verbal/oral consent procedure,

**Author Reponses**: Yes! The ethical committee was approved the oral consent procedure

b) Why written consent could not be obtained, and

**Author Reponses** This is because the study is just a qualitative research which did not have any interventions /clinical trial and did not have follow ups. In addition some of the participants were illiterate so taking oral consent was appropriate after giving a detail explanation on the aim of the study to the participants (see the detail of study information sheet in supporting information file 1).

c) How verbal/oral consent was recorded.

**Author Reponses**: What we’re doing is just we read the study information sheet which deals about the study objectives, the rights and confidentiality of the information obtained from participants, they were agreed to participate in the study then we included them to the study and we record their sayings (see the detail of study information in supporting information file 1) .

4. When reporting the results of qualitative research, we suggest consulting the COREQ guidelines: <http://intqhc.oxfordjournals.org/content/19/6/349>. In this case, please consider including more information on the number of interviewers, their training and characteristics; and please provide the interview guide used.

**Author Reponses**:

- We used COREQ guidelines in this qualitative study and we attached the interview guide as supporting information file 2&3
- There were two interviewers and two note takers who conducted the FGD and in-depth interviews. The interviewers are public health professionals who have master in public health specialties and had experience in qualitative research.

5. Please include a copy of Table 2 which you refer to in the Results section of your text.

**Author Reponses**: we are now put a copy of Table 2 based on the comment (see supporting information file _4)

6. Your ethics statement should only appear in the Methods section of your manuscript. If your ethics statement is written in any section besides the Methods, please delete it from any other section.

**Author Reponses**: edited/corrected based on the comment

II. **Point by point responses to the comments for reviewers**

**Comments to the Author**

1. Is the manuscript technically sound, and do the data support the conclusions?

   The manuscript must describe a technically sound piece of scientific research with data that supports the conclusions. Experiments must have been conducted rigorously, with appropriate controls, replication, and sample sizes. The conclusions must be drawn appropriately based on the data presented.

Reviewer #1: Partly

Reviewer #2: Yes

2. Has the statistical analysis been performed appropriately and rigorously?

Reviewer #1: N/A

Reviewer #2: Yes

3. Have the authors made all data underlying the findings in their manuscript fully available?

   The [PLOS Data policy](http://www.plosone.org/static/policies.action#sharing) requires authors to make all data underlying the findings described in their manuscript fully available without restriction, with rare exception (please refer to the Data Availability Statement in the manuscript PDF file). The data should be provided as part of the manuscript or its supporting information, or deposited to a public repository. For example, in addition to summary statistics, the data points behind means, medians and variance measures should be available. If there are restrictions on publicly sharing data—e.g. participant privacy or use of data from a third party—those must be specified.

Reviewer #1: No

Reviewer #2: Yes

4. Is the manuscript presented in an intelligible fashion and written in standard English?

   PLOS ONE does not copyedit accepted manuscripts, so the language in submitted articles must be clear, correct, and unambiguous. Any typographical or grammatical errors should be corrected at revision, so please note any specific errors here.

Reviewer #1: No

Reviewer #2: Yes

Review Comments to the Author

Please use the space provided to explain your answers to the questions above. You may also include additional comments for the author, including concerns about dual publication, research ethics, or publication ethics. (Please upload your review as an attachment if it exceeds 20,000 characters)

5. Reviewer #1: The quotes presented in this paper are very valuable, however, given that they are from nearly a year ago it would be useful to place them into context with the state of the pandemic now. Have the cases become unmanageable, likely as a result of the identified barriers, or was the government about to step up and provide a widespread educational campaign/provide mask, etc.? The paper, in particular the methods section, is hard to follow. The journal is likely able to help with grammar, but there are also several typos and the methods section is repetitive without making the recruitment strategy clear.

   For example:
   1. For the “source and study population” section, it is noted that “all people residing in South Gondar zone…were the source populations…” But I imagine individuals recruited for the in-depth interviews were patients in the clinic for the day? Either way the specific way they were targeted/recruited needs to be specified.

**Author Reponses**: individuals recruited for the in-depth interviews were not patients (it was editorial error) in the clinic for the day rather they were purposely selected community key informants (women health development armies, Health extension workers and religious leaders) for In-depth interviews while the FGD participants were selected purposely from community members with the help of local Kebele leaders.

2. Similarly, how were the members for the focus group recruited? While the sample size for qualitative studies is generally low, those in this study cover a large geographic region, large spread in age, and professions. For this reason, it would be useful to provide some kind of indication of who is saying what quote and potentially a table of frequencies per theme/category…maybe also separate this by community member vs. key informants.

**Author Reponses**:

The FGD participants were selected from the community members with the help of local leaders in the study sites. The study is conducted in one administrative zone (South Gondar zone) and it is not a regional study. The Themes, categories and the main findings /selected quotes were putted in the supporting information file based on the comment.

**(See supporting information file -4)**

3. Please provide the interview guide

Author Reponses: edited/corrected as commented, we provide the interview guide as **supporting information file 2&3**
4. Why was the interview guide created in English? Are there subtleties in the native language that would provide different meaning?

**Author Reponses**:

The guide (IDI & FGD) was first developed in English language and then translated to the local language (Amharic) to facilitate communication with participants. Interviewers and facilitators were fluent in written and spoken Amharic and English language.

6. Reviewer #2: Abstract: the last sentence of results is incomplete as same as the first one of conclusion.
   Abstract/.conclusion: don’t repeat results as conclusion

**Author Reponses**: edited/corrected based on the comments
Introduction: a short looking at current literature/previous studies improves the introduction.

**Author Reponses**: edited/corrected as commented
Methods: don’t use future verbs

**Author Reponses**: edited/corrected as commented
Methods: sampling method needs more exploration, it is to somewhat unclear in the current form.

**Author Reponses**: edited/corrected as commented
Methods: as the majority of participants were from rural regions, it should be notes as a limitation considering that the residential status may be a determinant of perceived barriers.

**Author Reponses**: edited/corrected as commented
Results: inserting a table including the main themes and sub-themes in the beginning of results is suggested.

**Author Reponses**: edited/corrected as commented (see supporting information file-4)
Discussion: the first 2 paragraphs of discussion should remove into introduction section. Start your discussion with brief presentation of main results.

**Author Reponses**: edited/corrected based on the comment

Discussion: discussion section should be improved by providing some policy recommendation/applications of results to deal with the problem and improve the status.

**Author Reponses**: edited/corrected based on the comment

7. PLOS authors have the option to publish the peer review history of their article ([what does this mean?](https://journals.plos.org/plosone/s/editorial-and-peer-review-process#loc-peer-review-history)). If published, this will include your full peer review and any attached files.

   If you choose “no”, your identity will remain anonymous but your review may still be made public.

   **Do you want your identity to be public for this peer review?** For information about this choice, including consent withdrawal, please see our [Privacy Policy](https://www.plos.org/privacy-policy).

Reviewer #1: No

Reviewer #2: **Yes:** Mohammad Amin Bahrami

[NOTE: If reviewer comments were submitted as an attachment file, they will be attached to this email and accessible via the submission site. Please log into your account, locate the manuscript record, and check for the action link "View Attachments". If this link does not appear, there are no attachment files.]

While revising your submission, please upload your figure files to the Preflight Analysis and Conversion Engine (PACE) digital diagnostic tool, <https://pacev2.apexcovantage.com/>. PACE helps ensure that figures meet PLOS requirements. To use PACE, you must first register as a user. Registration is free. Then, login and navigate to the UPLOAD tab, where you will find detailed instructions on how to use the tool. If you encounter any issues or have any questions when using PACE, please email PLOS at [figures@plos.org](mailto:figures@plos.org). Please note that Supporting Information files do not need this step.
