# Supplementary material for: Community risk perception and barriers for the practice of COVID-19 prevention measures in Northwest Ethiopia: A qualitative study
Source: PLoS One. 2021 Sep 24;16(9):e0257897. doi: 10.1371/journal.pone.0257897 (PMC8462701; doi:10.1371/journal.pone.0257897)
Supplement: Attachment — Submitted filename: Response to Reviewers.docx [file pone.0257897.s006.docx]

**June 8, 2021**

**Dear, Editor,**

**Greetings!**

Thank you very much for all the comments provided regarding our manuscript entitled “Community Risk perception and Barriers for the Practice of COVID-19 Prevention Measures in Northwest Ethiopia:  A Qualitative Study**”** which are fully accepted and included in the revised version. I have accordingly made necessary revisions on the paper following the comments and suggestions provided from the reviewer 1. For your kind consideration, please find a point by point response to the comments in the next page of this letter and a submitted new revised version of the manuscript.

All new changes have been highlighted in **dark blue** in the main document in order to facilitate review.

I hope that you will find the edits as per your expectation and I look forwards to hear from you
soon.

Yours Sincerely,

Aragaw Tesfaw (MPH, Epidemiology)

Lecturer, Department of Public health

College of health sciences, Debre Tabor University

Email: aragetesfa05@gmail.com

Phone: +251921743820

I. **Point by point responses to editor comments**

Journal Requirements:

- Please review your reference list to ensure that it is complete and correct. If you have cited papers that have been retracted, please include the rationale for doing so in the manuscript text, or remove these references and replace them with relevant current references. Any changes to the reference list should be mentioned in the rebuttal letter that accompanies your revised manuscript. If you need to cite a retracted article, indicate the article’s retracted status in the References list and also include a citation and full reference for the retraction notice.
- **Author Reponses**: We revised and checked the references accordingly based on the comment

Review Comments to the Author

Reviewer #1: Thanks for taking the time to update the language and methods that now much more clearly explain the qualitative approach. A number of additional grammatical suggestions are provided below for the abstract, introduction, and methods as well as additional questions/comments. The results section and discussion can still use updating to the language to clarify meaning. I make a few suggestions below for the results section specifically. At the moment the discussion mostly serves to re-summarize the results rather than place the findings in context of the ongoing pandemic. For example, can you more specifically state how these findings relate to other studies in Ethiopia, Africa, or other lower income countries? Coming from a public health group could you state a little more about how these findings could be used? Have other areas in the country/continent done a better job in implementing educational campaigns? Can you draw from other campaigns that have done a good job in communicating information to rural areas that may serve COVID-19? Could these findings drive approaches to the vaccine campaign once supplies become available? For example, how might religious beliefs impact the community from taking the vaccine?

**Author Reponses**: we would like to say thank you for the constructive comments forwarded from the authors which are fully acceptable and revised accordingly based on the suggestions and comments. We tried to edit the grammatical errors and language in each section of the document and we now made necessary revisions. We tried to improve also the discussion section by comparing other studies conducted in Africa and Ethiopia as suggested by the reviewer. We tried to mentioned areas in Ethiopia and Africa which done a better job in implementing educational campaigns regarding the prevention of COVID-19. Regarding the findings of our study, we also mentioned in the discussion section about its how our findings will be used. We tried to mention also other campaigns that have done a good job in communicating information to rural areas that may serve COVID-19. As the reviewer mentioned, our study findings can drive approaches to the vaccine campaign once supplies become available. As we mentioned in the main document, religious beliefs will have an impact the community from taking the vaccine, since we are currently facing the challenge even though there are limited vaccines in the country for high risk groups. Still people are reluctant to take the vaccine due to different perceptions. some studies on vaccine acceptance found that the willing ness to accept the vaccine in Ethiopia is low.
Specific comments
Abstract
Methods: Community key informants and health extension workers were selected purposely for in-depth interviews and focus group discussion to [should this be “on” instead of “to”] maintaining WHO recommendations for social distancing and use of appropriate personal protective equipment [delete the 's.]

**Author Reponses**: corrected based on the comments

Specifically state the sample size number in the abstract.
**Author Reponses**: edited/corrected based on the comments
FGD has not been defined in the abstract, this acronym is also not needed and could just state focus group discussions throughout the paper.
**Author Reponses**: edited/ corrected based on the comments

Results: Three main themes and five categories were emerged from… and 2 religious leaders were participated...[delete was] conducted among purposely selected community members. The age of the participants [delete was] ranged from 24 to 70 years…

**Author Reponses**: edited/corrected based on the comments

For the last two sentences I would rearrange them so that it states- The major identified barriers for practicing COVID-19 prevention measures were the presence of strong cultural and religious practices, perceiving that the disease does not affect the young, misinformation about the disease, and “the” lack of trust on the prevention measures.

**Author Reponses**: edited/corrected based on the comments

Conclusions: Socio-cultural, religious, and economics related barriers were identified from the participant’s narratives for the practice of COVID-19 prevention measures in south Gondar Zone. Our findings suggest the need to strengthen [delete “ing”] community


Background

The world is closely watching the outbreak of “the” respiratory illness…

The world is now suffering from this global pandemic which [has killed instead of “ kills”] millions
**Author Reponses**: edited/corrected based on the comments
I would suggest the following change for this thought given the widespread availability of vaccines in parts of the world-There are not any proven pharmacological treatments [delete “and vaccines” … “until now”] although a number of trials are [consider adding “currently underway by scientist across” and delete “tested worldwide by several scientists in”] the globe. [Consider adding… “While several effective vaccinations are now available and countries such as the USA and Israel have now vaccinated >50% of adults…something about availability in Ethiopia/Africa continent”]. However, there [delete “are” and add “continue to be” scientifically accepted and recommended prevention measures against COVID-19 infection such as social distancing, frequent hand washing, use of face mask, and proper use of alcohol-based hand rubs or sanitizers (6-8).
**Author Reponses**: edited/corrected based on the comments
With neither treatment nor vaccines [consider adding “readily available”], and [consider deleting “without preexisting immunity” because no one had preexisting immunity to this disease].

**Author Reponses**: edited/corrected based on the comments

For “the effect might be devastating” perhaps you mean, “the virus continues to threaten the country” because of the multiple health challenges the continent [do you mean the country?...if you mean continent you need to specifically state Africa]…[at the end of this paragraph you could add something about the recent spike in India as a reminder of the continued risk of this pandemic even when some parts of the world are returning to pre COVID levels of engagement]

To contain the pandemic, the country established a National Ministerial Committee and the government declared [should be a state of emergency and not the state of emergency] on April 8, 2020. [For the following sentence consider this rewording and numbering the elements…On March 16, 2020 the committee released guidelines that included: 1) 14 days quarantine of passengers (travelers?) in isolated centers; 2) Ethiopian Airlines to stop flying to 30 countries;… 9) temporarily ceasing (do you mean closing? ceasing means taking away from the owners) night clubs and bars; regulating market to avoid unethical exploitation of the situation

In this study, healthcare workers and key informants in the community including local leaders [were not instead of will be] interviewed

The following sentence is unclear/can you update what you are trying to say here-This is helpful from the view of the following perspectives. Healthcare workers the frontline in battling the spread infection as a whole (12) . Secondly, community leaders are also the right of the country in prevention the COVID-19 infection (13).

**Author Reponses**: edited/corrected based on the comment in the main document
[Can remove the following sentence because it repeats points noted in the prior section, if desired to keep could say something like.. “The basic protective measures against COVID-19 including distancing, personal protective equipment, and handwashing as noted above are poorly practiced in the community of …] Along with the above scientific explanations, controlling the spread of infection is essential to preventing outbreaks of COVID-19 (14).
**Author Reponses**: edited/corrected
Consider this rewording-Basic protective measures against the new coronavirus including stay at home, frequently washing hands with soap and water using alcohol-based hand rub or wash…Thus, the implementation of these practices to reduce the spread of COVID-19 would be challenging in Ethiopia…

RECOMMENDED [RECOMMENDED has not been previously defined or presented this way] prevention measures among community key informants and other community member’s [delete “perspective”] in South Gondar Zone, North west [Northwest?] Ethiopia.

**Author Reponses**: edited/corrected in the main document
Methods

Study area and period

Can you define Woredas?

**Author Reponses**: Woredas are the lower administrative areas next to zone called also districts according to the Ethiopian government structure. A number of woreda’s form a zone in combination. Kebele $\to$Woreda$\to$ Zone $\to$ Region $\to$Federal

This study was conducted on purposely selecting [do you mean selected?, but “purposely selecting could be removed either way”]
**Author Reponses**: edited/corrected based on the comment
Currently, the zone is hardly working…[this statement is too subjective, can it be made more objective by stating how many beds the isolation center/ quarantine center/treatment center is able to accommodate. Similarly, later in the paragraph the 150 cases doesn’t seem like much, but could have more weight if clear that the area would not be able to handle a surge of cases].
**Author Reponses**: edited/corrected based on the comment

Participants of the study and sampling procedures

…from the community [delete “were”] participated [in instead of for] Focus Group Discussions.
**Author Reponses**: edited/corrected based on the comment
Data collection tools and procedures

[Can remove this sentence as it is stated above]-The interview process was continued until the data reach saturation, which defined as the point when answers no longer provide new or additional information on the research question and when recurrent patterns became apparent in the participants descriptions.

Some additional “were/was” before “participated/enhanced” that need to be removed
**Author Reponses**: edited/corrected based on the comment
Results
Table 1-Need to keep the use of capital letters consistent

For quotes can you specifically state which IDI participant provided the quote, so which career were they coming from
**Author Reponses**: edited/corrected based on the comment
The non-quoted paragraphs of “Sociodemographic and economic related barriers” section were confusing, please reword.
**Author Reponses**: edited/corrected based on the comment
“Cultural religious related barriers”
What does this mean? “…did not affect those who had a strong religious practice”

**Author Reponses**: Cultural and religious barriers were the major perceived barriers mentioned from the participants of the study since there are strong religious and cultural practice in the area. Most people trusts religious rules than the modern health care practice. their trusts on the prevention methods is low.

As the participants said, almost all participants [delete “were”] trusted on the use of traditional treatments [delete “which will be”] prepared from different herbal plants. [Delete “As the” participants described, the disease [as the instead of “is like”] common cold and [believed it could] be healed by common traditional treatment modalities of the common cold. When [delete “they were”, “also”, “they said as they will use these traditional treatments prepared by the local traditional healers from different plant leaves and roots and to use holy water.” [the last part is redundant]

**Author Reponses**: we removed /corrected based on the comment

- Reviewer #2: thanks to authors, all of my comments have been addressed. The manuscript can accepted in current version.

**Author response:** thank you very much for reviewing and giving constructive comments
