# Supplementary material for: Community risk perception and barriers for the practice of COVID-19 prevention measures in Northwest Ethiopia: A qualitative study
Source: PLoS One. 2021 Sep 24;16(9):e0257897. doi: 10.1371/journal.pone.0257897 (PMC8462701; doi:10.1371/journal.pone.0257897)
Supplement: Attachment — Submitted filename: Response to Reviewers.docx [file pone.0257897.s007.docx]

**September 06, 2021**

**Dear, Editor,**

**Greetings!**

Thank you very much for all the comments provided regarding our manuscript entitled “Community Risk perception and Barriers for the Practice of COVID-19 Prevention Measures in Northwest Ethiopia:  A Qualitative Study**”** which are fully accepted and included in the revised version. I have accordingly made necessary revisions on the paper following the comments and suggestions provided from the reviewer. For your kind consideration, please find a point by point response to the comments in the next page of this letter and a submitted new revised version of the manuscript.

All new changes have been highlighted in **dark blue** in the main document in order to facilitate review.

I hope that you will find the edits as per your expectation and I look forwards to hear from you
soon.

Yours Sincerely,

Aragaw Tesfaw (MPH/Epidemiology)

Assistant Professor, Department of Public health

College of health sciences, Debre Tabor University

Email: aragetesfa05@gmail.com

Phone: +251921743820

I. **Point by point responses to reviewer comments**

Reviewer #1: Thank you once more for taking the time to address my prior comments. A number of grammatical errors remain especially in the discussion which makes the paper at times hard to read. Reading items aloud can sometimes help with these issues. Some suggestions below:

In the abstract, methods: “A total of 23 people were participated in the study” were should be removed.

**Author response: thank you very much for reviewing and giving constructive comments. We removed as commented by the reviewer**

Similarly in the results of the abstract: Were should be removed in the following sentences… “Three main themes and five categories were emerged”… “and 2 religious leaders were participated”…

Abstract, results: … “(7 participants in each round) was”… was should be were

abstract, conclusions: … removed the “s” from economics

**Author response: corrected based on the comment in the main document**

Background: Update the following sentence “There are no any proven pharmacological treatments developed for the disease until now, although a number of trials are currently underway by scientists across the globe. However, COVID-19 vaccines are developed within the shortest period in the history of vaccine production and now become now available and countries such as” to…There are no pharmacological treatments developed for the disease, although a number of trials are currently underway by scientists across the globe. However, COVID-19 vaccines are now available and countries such as…” (The other text is not needed and can be misleading without additional context about the time of vaccine development).

**Author response: corrected based on the comment**

Background: Please update the following sentence… “…pre-COvid levels of engagement, the risk of the pandemic is continued and becomes a global challenge (12). According to the June 8, 2021 Worldometer report, currently India and South Africa…” to …pre-COVID levels of engagement, the risk of the pandemic has continued and is a global challenge (12). According to the June 8, 2021 Worldometer report, India and South Africa…
**Author response: Updated based on the comment in the main document**

Background: When listing out the guidelines make sure you are using a “;” after the guideline and not a “:” as you are before 3) and 4). Similarly, you are missing some numbers. There should be a “8” before medical professional training, which will require you to renumber afterwards. And “support the regions preparedness…” will be number 12. Consider the following rewording for the rest of that section. In this study, healthcare workers and key informants in the community including local leaders were interviewed either individually or as part of focus groups to share their perceived barriers to COVID-19 prevention measures. This is helpful from the view of the following perspectives. Healthcare workers are the frontline in battling the spread of infection as a whole (16)… [for the last sentence what does “right of the country” mean? Consider alternative wording for clarity].

**Author response: corrected based on the comment in the main document**

In the next paragraph be sure to capitalize “South Gondar Zone” for consistency
**Author response: corrected/edited based on the comment**

Methods: For the data analysis procedure section, did you name the open code software?
**Author response:** open code software version 4.02 /**corrected based on the comment in the main document**

Methods, ethical approval and consent to participant: Update… “This is because the study is just a qualitative research which did not have any intervention /clinical trial and did not have follow-ups. In addition, some of the participants were illiterate…” to This level of consent was sufficient because the study was not an intervention/clinical trial and did not have follow-ups. In addition, some of the participants were illiterate…

Results: In addition to some grammar errors and reworking of some sentences that could be shortened for clarity, some work could be done to better organize the quotes. For example, temperature was noted both under Socio-demographic and economic related barriers and Cultural and religious related barriers.

**Author response: corrected based on the comment in the main document**
Discussion: Consider the following updates… As evidence shows, the recommended prevention methods of COVID-19 are effective measures to control the spread of the pandemic (10, 14, 23). However, the practice of such prevention measures is not consistent regionally as documented in Ethiopia despite efforts are made by the government (11, 15, 24). Therefore, we examined the perceived barriers for the practice of prevention measures and community risk perception of COIVID-19 focusing on both rural and urban settings.
**Author response: corrected based on the comment in the main document**
Discussion: For reference 25 you note “less than half percent: which would mean 0.5%...I believe you mean to say less than 50 percent. As the abstract notes “The overall rate of information exposure about COVID-19 was 44.9%.”

**Author response: thank you! we corrected based on the comment in the main document**
Discussion: Consider the following rewrite for paragraph 2- The other important recommended and effective prevention measure is stay at home; however, our findings showed that it is a very difficult preventive measure to practice since most people live a hand--to--mouth way of life. Therefore, they need to get their daily consumption through daily work otherwise many will face hunger. As a result, most people cannot stay at home. Further, because Ethiopia is one of the lowest-income countries in the world and has a very young population, many people live together in a clustered environment, approximately 4-8 people in a single household [note, I don’t think it uncommon for 4 people to live in a single household so maybe you mean to additional note in a single room? Or note the size of the household]. This living situation provides a ripe environment for a contagious diseases like COVID-19. [There is also a sentence about negligence in your text but I’m not sure how the negligence part fits with the above text].

Again, there are several other sections within the discussion that could be updated for clarity such the increased interactions because of religious and cultural practices. The latter is an important point, again one could think back to India’s surge in relation to their religious celebrations or the number of mass outbreaks in the United States as a result of church attendance and singing without masks, but the sentences need to be rewritten to make the point more salient.

**Author response:** thank you very much for reviewing and giving constructive comments, we tried to modified the document based on the comments
